# Gross ecosystem product (GEP): Quantifying nature for environmental and economic policy innovation

**DOI:** 10.1007/s13280-023-01948-8

**Published:** 2023-11-09

**Authors:** Hua Zheng, Tong Wu, Zhiyun Ouyang, Stephen Polasky, Mary Ruckelshaus, Lijuan Wang, Yi Xiao, Xiaolong Gao, Cong Li, Gretchen C. Daily

**Affiliations:** 1 State Key Laboratory of Urban and Regional Ecology, Research Center for Eco-Environmental Sciences, Chinese Academy of Sciences, No. 18 Shuangqing Road, Haidian District, Beijing, 100085 China; 2 University of Chinese Academy of Sciences, Beijing, 100049 China; 3 Natural Capital Project, Stanford University, Stanford, CA 94305 USA; 4 Department of Applied Economics, University of Minnesota, 1994 Buford Avenue, St. Paul, MN 55108 USA; 5 Natural Capital Project, University of Minnesota, St. Paul, MN 55108 USA; 6 School of Economics and Finance, Xi’an Jiaotong University, No.74 West Yanta Road, Yanta District, Xi’an, 710061 China; 7 Department of Biological Sciences and Woods Institute for the Environment, Stanford University, Stanford, CA 94305 USA

**Keywords:** Ecosystem accounting, Ecosystem assets, Ecosystem services, Gross ecosystem product, Nature’s contribution to people, Valuing nature

## Abstract

The large-scale loss of ecosystem assets around the world, and the resultant reduction in the provision of nature’s benefits to people, underscores the urgent need for better metrics of ecological performance as well as their integration into decision-making. Gross ecosystem product (GEP) is a measure of the aggregate monetary value of final ecosystem-related goods and services in a specific area and for a given accounting period. GEP accounting captures the use of many ecosystem services in production processes across the economy, which are then valued in terms of their benefits to society. GEP has five key elements that make it transparent, trackable, and readily understandable: (1) a focus on nature’s contributions to people; (2) the measurement of ecosystem assets as stocks and ecosystem services as flows; (3) the quantification of ecosystem service use; (4) an understanding of ecosystem service supply chains through value realization; and (5) the disaggregation of benefits across groups. Correspondingly, a series of innovative policies based on GEP have been designed and implemented in China. The theoretical and practical lessons provided by these experiences can support continued policy innovation for green and inclusive development around the world.

## Introduction

As conventionally measured by gross domestic product (GDP) in constant dollars terms, the world economy more than doubled between 1990 and 2022 (World Bank 2023). However, the world’s stocks of ecosystem assets—such as forests, grassland, wetlands, fertile soils, and biodiversity—and their flows of ecosystem services have come under increasing stress and disturbance (Ouyang et al. 2020). The loss and degradation of ecosystem assets significantly impairs the resilience and sustainability of ecosystem services and consequently poses a threat to economic activity and human well-being (MA 2005; Rockström et al. 2009; IPCC 2018; Diaz et al. 2018; IUCN 2019; IPBES 2019; Polasky et al. 2019). There has been growing recognition in recent years that the way we measure development and well-being has tremendous shortcomings demanding (e.g., Stiglitz et al. 2010).

In particular, there have been growing calls to better account for the contribution of nature to human well-being—that is, to mainstream ecosystem services into decision-making (MA 2005; Ouyang et al. 2020). Over the past three decades, the economic value of specific ecosystem types—such as forests, wetlands, mountains, coasts, and lakes—and specific ecosystem services—such as pollination (Ricketts et al. 2004; Breeze et al. 2016; Matias et al. 2017), pest control (Cleveland et al. 2006; Karp et al. 2013; Zhang et al. 2018), and water purification (Keeler et al. 2012; Zheng et al. 2013)—have been assessed at the watershed, regional, and even global scales (de Groot et al. 2012; Rao et al. 2015; Reynaud and Lanzanova 2017; Chaplin-Kramer et al. 2019; Davidson et al. 2019; Gret-Regamey and Weibel 2020; Jiang et al. 2021b; Taye et al. 2021). These studies have contributed to a global movement connecting ecology with economics, conservation with development (Mandle et al. 2019; D’Amato et al. 2020; Ding et al. 2022).

In 2012, the United Nations approved a global framework called the System of Environmental–Economic Accounting (SEEA) (see https://seea.un.org and http://go.nature.com/38lc38h). Since then, several initiatives have built environmental-economic accounts using the SEEA framework, including the United Nations Statistics Division’s Natural Capital Accounting and Valuation of Ecosystem Services project and the Wealth Accounting and Valuation of Ecosystem Services partnership led by the World Bank. This approach has also recently been applied at the country scale (Banerjee et al. 2019a, b). Relatedly, the World Bank has sought to measure the Changing Wealth of Nations (World Bank 2021), aiming to incorporate ecosystem assets alongside more conventional forms of capital, and various groups have pursued similar approaches to quantify inclusive/comprehensive wealth (Hamilton and Clemens 1999; World Bank 2006, 2011; Arrow et al. 2012; United Nations University et al. 2012, 2014; Polasky et al. 2015; Managi and Kumar 2018). However, the goal of simultaneously accounting of ecosystem assets (which are stocks) and the services they provide (which are flows), and then to distinguish the supply from the use of ecosystem services, has remained a conceptual, methodological, and practical challenge (Maes et al. 2018; Vallecillo et al. 2020). In particular, while ecosystem services or natural capital accounting has been conducted across the world, the question of how to move from accounting knowledge to action remains largely unanswered (Daily and Ruckelshaus 2022).

Because of China’s economic scale and complexity, along with the challenging dynamic between its rapid growth and severe ecological impacts, innovative approaches have been developed to mainstream ecosystem services into decision-making. The need to protect and restore ecosystem assets and enhance the flow of ecosystem services has been acknowledged at the highest levels of government. For instance, in a widely cited speech to the 19th National Congress of the ruling Communist Party of China (CPC), President Xi Jinping averred that, “Lucid waters and lush mountains are invaluable assets” (The Chinese Government 2015). To advance this ideal of sustainability, scholars put forward the concept of gross ecosystem product (GEP) to account for nature’s contribution to people (Ouyang et al. 2013).

GEP builds on prior work that have developed integrated environmental-economic accounts, including the UN SEEA (United Nations et al. 2012) and the SEEA Experimental Ecosystem Accounting framework (EEA) (United Nations et al. 2013). In the latest version of the SEEA-EA, the concept of GEP was included as a potential indicator to account for ecosystem services flows in monetary terms (UN 2021). GEP is now being implemented in a variety of decision-making contexts across China and several other countries such as Colombia, Sri Lanka, and Sweden, which are planning to engage in GEP accounting. This paper will (i) introduce the GEP concept, including its principles and accounting methods and (ii) summarize the real-world experiences of applying GEP to support environmental policy innovation. These lessons can help inform the scaling-up of local and national successes in natural capital stewardship to the global scale.

## The GEP concept and its defining characteristics

**Fig. 1:**
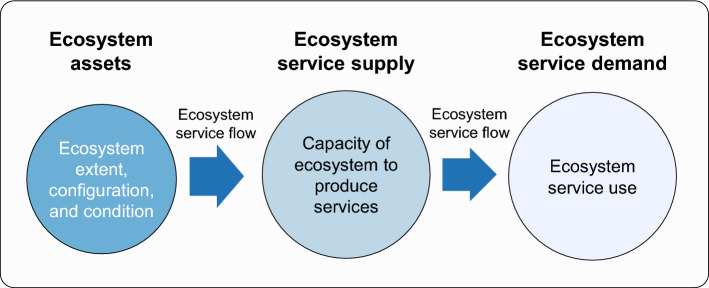
Relationships between ecosystem assets, ecosystem service supplies, and ecosystem services use (Modified from Maes et al. 2018)

Stocks of ecosystem assets are the material basis for the resilience and sustainability of ecosystem service flows (Ouyang et al. 2020; Vári et al. 2022). Ecosystem assets, as measured by ecosystem extent, configuration, and condition, generate ecosystem service supply (i.e., ecosystem service capacity). When different stakeholders at different scales (e.g., local, regional, global) make specific demands (e.g., for food, water, health, security) (MA 2005), they rely on ecosystem service supplies to realize social benefits. Ecosystem service supplies are partially and fully consumed by people, thereby becoming utilized ecosystem services (Maes et al. 2018) (Fig. 1 and Box 1). The aggregate monetary value of these utilized ecosystem services in a given region and accounting period is GEP (Ouyang et al. 2013, 2020). Here, ecosystem services can be classified into material services (the contribution of nature to the provision of food, water supply, and so forth), regulating services (the contribution of nature to carbon sequestration, flood mitigation, soil retention, sandstorm prevention, and so forth), and nonmaterial services (the contribution of nature to eco-tourism, nature experience for mental health, and so forth) (Diaz et al. 2018).

**Fig. 2:**
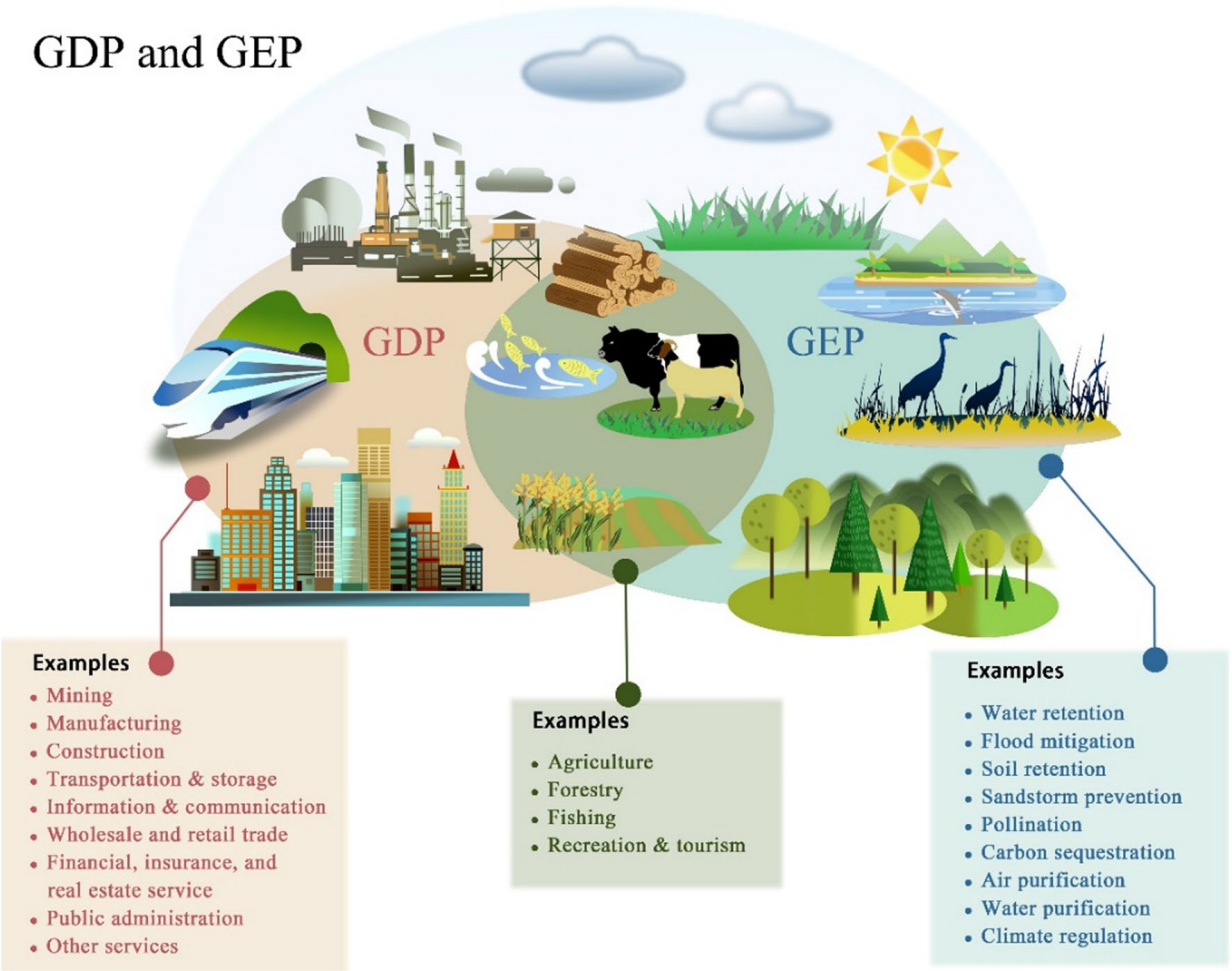
Relationship between GDP and GEP: complementary but overlapping measures

GEP is a measure that translates ecosystems’ contributions to people into economic and monetary terms, which is fundamental to assessing the actual use and valuation of ecosystem services. Analogous to GDP, GEP uses market prices and surrogates for market prices to calculate the accounting value of ecosystem services. These are then aggregated into a measure of the contribution of ecosystems to the economy. The power of GEP is enhanced through the use of similar methods as those underpinning GDP. GEP can be a useful complement to GDP, highlighting the contribution of nature overlooked in GDP calculations. It is important to note the overlap between GEP and GDP (Fig. 2), since some ecosystem service outputs included in GEP are also inputs into the production of the goods and services measured in GDP (e.g., agricultural products, timber, eco-tourism). For this reason, one cannot simply sum GEP and GDP and generate a meaningful value.

In summary, GEP is an innovative contribution to the knowledge foundation and practice relating to ecosystem services and natural capital in two primary ways. First, GEP is a novel aggregate measure of the value of utilized ecosystem services, which summarizes key contributions that nature makes to the economy (Ouyang et al. 2013; Ma et al. 2015; Ouyang and Jin 2017). Second, the principles and strict definitions applied to GEP—to be discussed subsequently—provide clear linkages with decision-making and have demonstrated broad policy applicability in China, and can also be applied in other countries. GEP is a lower-bound measure of the contributions of ecosystems to society because people are completely interdependent with nature in ways that cannot be captured meaningfully in economic terms (e.g., Goulder and Kennedy 1997; Kimmerer 2013). Yet GEP offers a powerful approach to transform the economic system to recognize key values that are presently invisible in decision-making.

Box 1. Several related concepts for GEP accounting

- **Ecosystem assets:** stocks referring to the ecosystems that provide services that support human well-being (Ouyang et al. 2016a; Song et al. 2019). Comprehensive information is needed on the extent (area), spatial configuration, and condition (quality) of ecosystems (e.g., forest, grassland, wetland, and farmland).
- **Ecosystem service flow:** the process of ecosystem services going from the provisioning area and its ecosystem stewards to the beneficiary areas and their consumers (Wang et al. 2022).
- **Ecosystem service supply:** an ecosystem’s theoretical capacity to produce services, which is determined by ecosystem structure and function (Wang et al. 2022).
- **Ecosystem service use:** the amount of produced ecosystem services that reaches and benefits people.

## GEP accounting methods

**Fig. 3:**
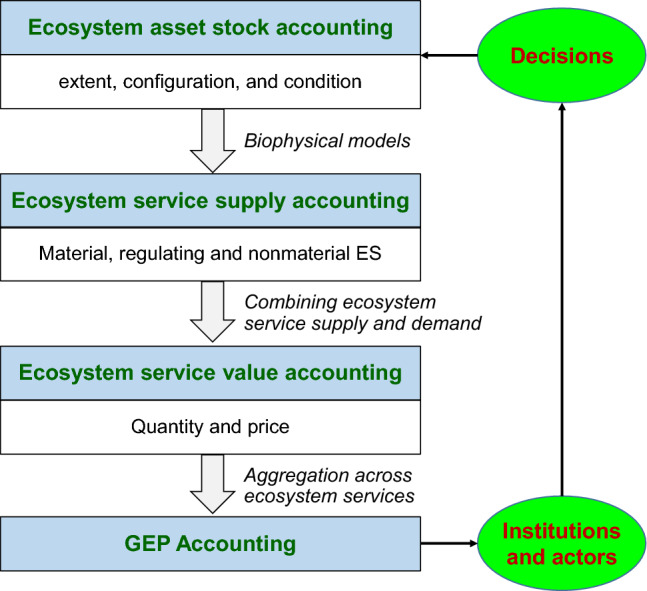
GEP accounting processes in the context of decision-making

Four steps are needed to understand and properly assess GEP: (i) ecosystem asset stock accounting; (ii) ecosystem service supply accounting; (iii) combining supply and demand for ecosystem service quantity and price accounting (which determines the value of each ecosystem service); and (iv) aggregation across value of ecosystem services for GEP accounting (Fig. 3). Ouyang et al. (2020) combine data on ecosystem assets, recent advances in ecosystem services modeling, and combining supply with demand to determine quantity and price, to show that the GEP accounting can be done using existing data.

A complete environmental-economic accounting systems, for instance as envisioned in SEEA, would include measures of the value of the flow of ecosystem services (GEP) along with the value of ecosystem stocks (a measure of wealth). Measuring the value of ecosystem assets is important for sustainability. In fact, maintaining or increasing the value of inclusive wealth, which is a measure of the value of all assets that includes natural capital (which includes ecosystem assets), manufactured capital, human capital, and social capital, is a measure of sustainability (Arrow et al. 2012; Polasky et al. 2015). Measuring the value of ecosystem assets, however, is challenging. One method to value ecosystem assets is to measure the value of ecosystem services they generate and use the income-capitalization-method (Bo et al. 2017). In what follows, we focus on accurately assessing the value of ecosystem services and aggregating these values into GEP, which requires ecosystem asset accounting but not assessing the value of ecosystem assets.

### Ecosystem asset stock accounting

Sustainable flows of ecosystem services depend upon maintaining the stock of ecosystem assets (in terms of extent, configuration, and quality). Ecosystem assets include natural ecosystem assets (e.g., forests, shrublands, grasslands, rivers, and lakes) and modified ecosystem assets (e.g., farmland and reservoirs) (Ouyang et al. 2016b; Vackaru and Grammatikopoulou 2019). In contrast to some conventional definitions of “natural capital,” ecosystem assets do not include coal, oil, natural gas, and other abiotic resources that cannot be regenerated over a practical timeframe (Turner and Daily 2008; Ouyang et al. 2016b). The evaluation of ecosystem assets encompasses analysis of the extent (e.g., the areas covered by different ecosystem types) and condition (e.g., biomass, water quality, vegetation coverage) of ecosystems, as well as the integration of these factors. Field surveys and remote sensing are two of the most commonly used ways to study the status and trends of ecosystem assets (Huang et al. 2020).

### Ecosystem service supply accounting

Ecosystem service supply focuses on the biophysical production function of ecosystems services. A series of biophysical models and software, such as the Integrated Valuation of Ecosystem Services and Tradeoffs (InVEST) (Sharp et al. 2018), or the Soil and Water Assessment Tool (SWAT) (Arnold et al. 2012), can be used to assess ecosystem service supply. Ecosystem service supply can be quantified by applying these models to data on ecosystem assets (as determined in step 1) and additional biophysical information (e.g., soil, slope, climate data). Accurate and spatially explicit parameters play a key role in assessing ecosystem service supply. Ecosystem surveys conducted within a unified temporal-spatial scale are essential for accurate accounting.

### Ecosystem service value accounting

Combining supply of an ecosystem service (as determined in step 2) with demand for an ecosystem service can be used to determine ecosystem service quantity (amount of ecosystem service actually used), and price for an ecosystem service. Ecosystem service demand is defined as the willingness-to-pay for an ecosystem service and represents the contribution of an ecosystem service to human well-being expressed in monetary terms. Multiplying ecosystem service quantity by price determines ecosystem service value.

To maintain consistency in the accounting of ecosystem services (so that different services can be aggregated using a common unit in step 4), monetary valuation in GEP is based on exchange price—prices at which goods and services are exchanged in markets, or estimation of exchange prices for non-market goods and services. Monetary values in GEP can therefore potentially be used to analyze the contribution of nature to the economy for many purposes (Hein et al. 2020), such as green growth evaluation, benefit–cost analysis, payments for ecosystem services, and the development of GEP-based financing mechanisms.

Where available, data on market prices are used. In cases where market prices for ecosystem services are not available, the price may be estimated by using market prices in related markets. For example, the hedonic property price method uses property prices to estimate the value of environmental amenities (Freeman et al. 2014). Prices of final goods that combine ecosystem services and other inputs can be used to estimate the value of ecosystem services by subtracting the value (cost) of other inputs from the value of the final good (Ouyang et al. 2020). In addition, other revealed and stated preference methods are available to use to estimate non-market values (Freeman et al. 2014). The UN Statistics Division has recommendations on pricing methods for different ecosystem services (UN 2021).

In some cases, the price of an ecosystem service will be zero, as for example where an ecosystem provides a service that is not used by anyone (e.g., water purification is an uninhabited watershed). Even when there is demand for an ecosystem service, the price will be zero when demand for the service is less than supply when price is zero. For example, vegetation in ecosystems produces oxygen that is essential for human life. However, the supply of oxygen in the atmosphere is currently so large that it exceeds demand even when price is zero.

By using readily calculable ecosystem service accounting prices, which presently represents the most challenging step in accounting, GEP provides a tractable approach for bringing ecosystem services—including those that are not marketed—into decision-making processes and institutions.

### Aggregation into GEP

The last step in GEP accounting is to combine the values of different ecosystem services into an aggregate measure of GEP, which is defined as:

$$\text{GEP}=\sum_{i\in I}{\gamma }_{i}{p}_{i}{q}_{i}$$

where *I* is the set of ecosystem services, γ_i_ is the proportion of the accounting value of a given ecosystem service attributable to nature, *p*_*i*_ is the accounting price of ecosystem service *i*, and *q*_*i*_ is the quantity of ecosystem service *i*. For regulating ecosystem services, the entire value of a given service is attributable to nature (*γ*_*i*_ = 1). For other services, including many material services, there are contributions from human labor and human-made inputs, so *γ*_*i*_ < 1. In aggregating ecosystem service values, care must be taken to avoid double-counting of values. For example, including both the value of pollination services and the value of crop production attributable to nature would double count the contribution of pollinators to the value of crop production.

## The application of GEP in policy

**Fig. 4:**
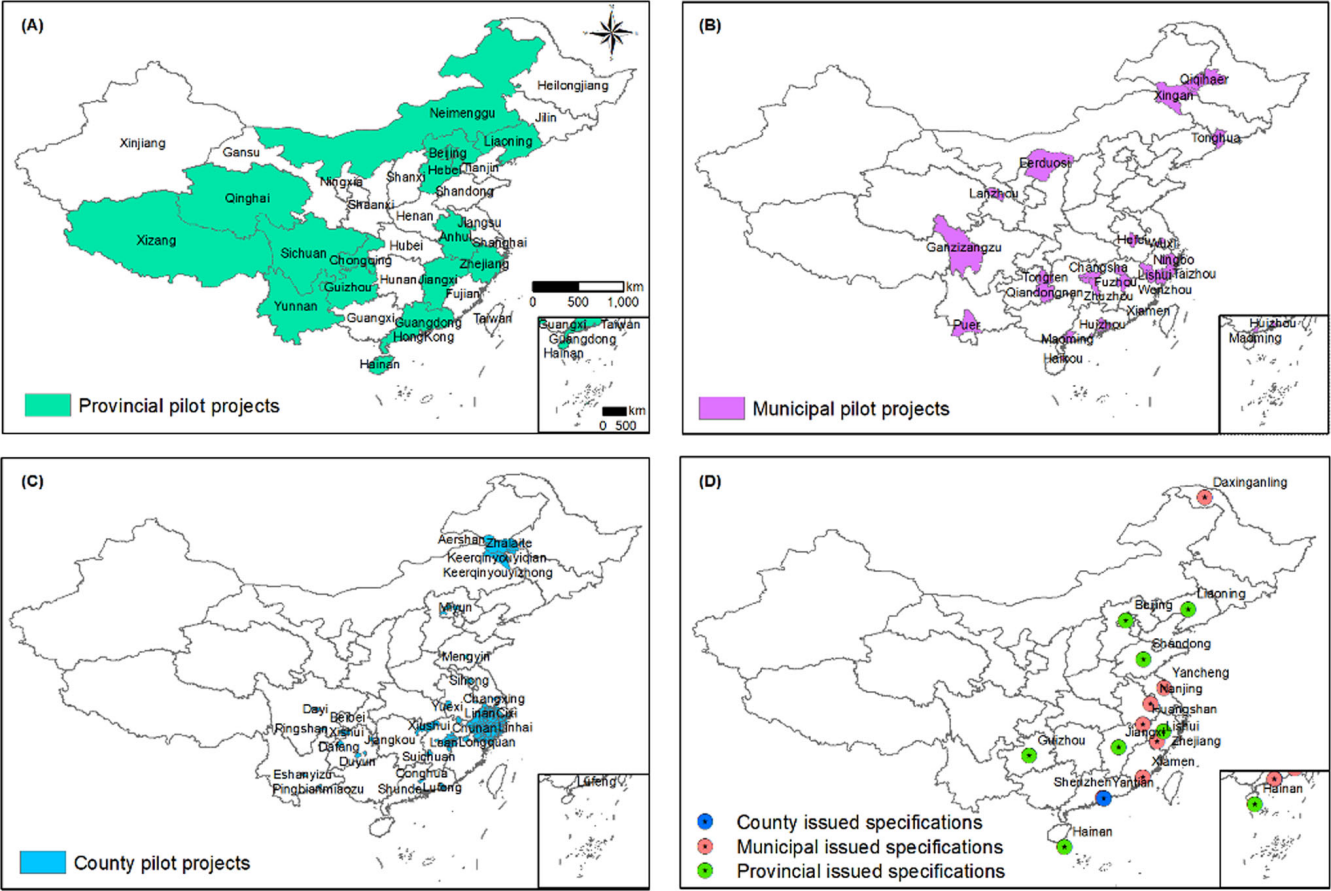
GEP accounting pilot projects and official accounting specifications in China at the provincial, municipal, and county levels. **A**–**C** refer to projects at the provincial, municipal, and county levels, respectively. **D** refers to the accounting specifications published by administrative level

There are now numerous applications of GEP accounting. As of the writing of this paper, China has conducted at least 196 GEP projects at different scales including 16, 29, and 151 at the provincial, municipal, and county levels, respectively (Fig. 4A–C). To improve GEP accounting for different administrative regions and in different geographical, climatic, and cultural settings, China has issued a total of 16 GEP accounting technical specifications/guidelines (Fig. 4D), with one at the national level and 15 at the local level. In particular, the National Development and Reform Commission and the National Statistics Bureau of China jointly released GEP accounting standards (“Ecosystem Assessment Guidelines for Gross Ecosystem Product Accounting”) (NDRC and NSB 2022) to standardize approaches across the country. Of the 15 local specifications, seven were issued at the provincial level, seven at the municipal level, and one at the county level (Fig. 4). Differences in the abovementioned GEP accounting technical specifications are mainly in terms of the indicator system, accounting parameters, and pricing methods—which depend on data accessibility. The specification for a given region allows GEP accounting results to be intertemporally comparable.

**Fig. 5:**
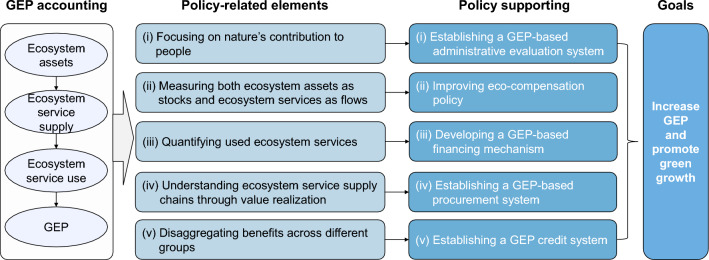
GEP accounting, policy-related elements, and applications in policy

The concept, principles, strict definitions, and accounting components of GEP provide a transparent, trackable, and easily understandable way to integrate the value of ecosystem assets as well as ecosystem services into decision-making processes. These elements also provide operational pathways for supporting green and inclusive development at various scales. Five following policy-related elements are emphasized in GEP (Fig. 5).

The first element is focusing on nature's contribution to people. GEP focuses on nature’s contribution to people, which provide an important indicator for integrating the ecological determinants of human well-being into economic development. The indicator can be used to support policy evaluation as well as the standardization of government or enterprise performance, in order to guide them toward increasing nature’s contributions to people.

The second element is measuring both ecosystem assets as stocks and ecosystem services as flows. Ecosystem asset stocks and ecosystem services flows are simultaneously assessed in GEP accounting. It is thereby feasible to acquire relevant information on the providers of ecosystem services and the quantity of ecosystem service use delivered to beneficiaries. This information is crucial for the evaluation of eco-compensation (i.e., payments for ecosystem services), in terms of identification of recipients, allocation of funds, and overall effectiveness.

The third element is quantifying used ecosystem services. GEP measures ecosystem service use in monetary terms. This opens channels between GEP accounting and cost–benefit analysis as well as toward potential market transactions. Correspondingly, ecosystem asset transactions and GEP-based green financing mechanisms have been developed (as will be discussed subsequently).

The fourth element is understanding ecosystem service supply chains through value realization. Ecosystem assets generate ecosystem service supplies, which then are utilized in whole or in part by society and thereby translated into ecosystem service use. A transparent and traceable supply chain produces three key pieces of information for policy: (i) where ecosystem services are generated; (ii) how much ecosystem service supply produced, and (iii) the quantity of utilized ecosystem services, including who actually benefits from them. This information can delineate, for example, the roles played by rural land stewards, governments, and enterprises, which can inform procurement, zoning, and other forms of policy.

The fifth element is disaggregating benefits across different groups. Each ecosystem service has one or multiple beneficiaries at various spatial scales. GEP can clearly link the beneficiaries and stakeholders of ecosystem service use at the local, regional, and global scales, and correspondingly disaggregate benefits across different groups. For example, the beneficiaries of the water retention service flow can be disaggregated into residents, enterprises, and farmers at specific spatial scales. It can also be disaggregated into stakeholders at the local, watershed, and regional scales. Disaggregating beneficiaries and stakeholders at different spatial scales provides information for the development of financing and credit systems for ecological protection and natural capital-based economic development.

China has advanced a series of innovative policies by synthesizing lessons from existing applications of GEP accounting as well as earlier examples of effective ecosystem services management and payment schemes (Arkema et al. 2015; Olander et al. 2017; Rieb et al. 2017; Van Wensem et al. 2017; Mastrángelo et al. 2019; Mandle et al. 2021). Five of these policies are detailed below (Fig. 5).

### Establishing a GEP-based administrative evaluation system

As a useful complement to GDP, GEP highlights the contribution of nature overlooked in GDP calculations. GEP accounting can be incorporated into the performance evaluation system of administrators to guide development that incentivizes ecosystem protection and restoration and to promote green, inclusive development.

#### GEP application mechanism

**Fig. 6:**
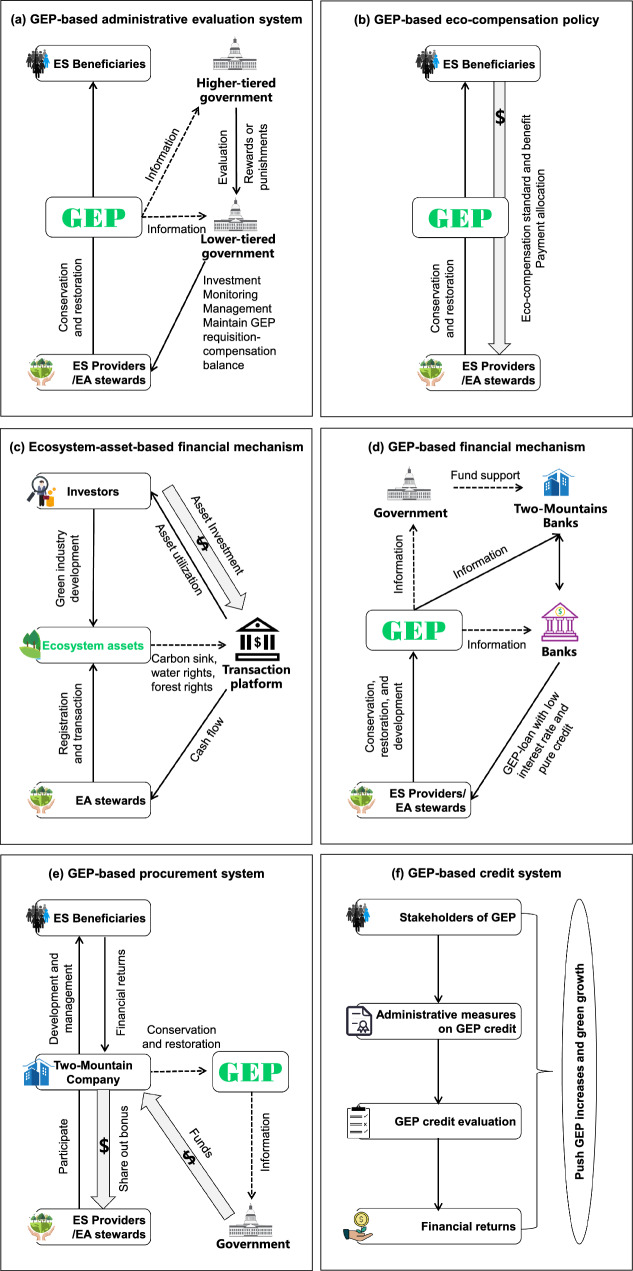
Policy application typology of GEP and ecosystem asset (EA) in China

Both higher-tiered and lower-tiered governments, alongside ecosystem service providers (often land stewards), are involved in GEP-based systems of administration evaluation. The higher-tiered government (e.g., city level) uses GEP to evaluate the performance of lower-tiered administrators (e.g., at the county level), who are directly responsible for governing ecosystem service providers (Fig. 6a). In many administrative regions (e.g., the cities of Lishui, Shenzhen, and Pu’er, and Ezhou), the government has clarified the responsibility of their lower-tiered counterparts (e.g., counties) and their constituent departments for enhancing GEP.^1^ Both GEP and GDP growth have become binding performance metrics in the evaluation system for a number of counties. GEP is also being used to audit the performance of officials leaving their posts (Lan and Liu 2022), serving as a benchmark for promotion or retirement prospects. During evaluation processes, the GEP improvement target is usually set every 5 years, and the administrative evaluation of progress toward that goal is disclosed to the public annually.

#### Effectiveness

The incorporation of GEP into administrative evaluation has institutionalized the incentive to advance ecosystem protection and restoration (e.g., in Lishui).^2^ The GEP-GDP dual growth mandate has guided the city for achieving improvements in both indicators (e.g., in Shenzhen).^3^ After institutionalizing GEP performance evaluation, the higher-tiered government (at the city level) can correspondingly implement reward and punishment mechanisms to spur investment in natural capital, with GEP and its growth rate serving as the reference standard. Furthermore, a GEP-based mechanism to balance requisition and compensation was established in certain regions to incentivize lower-tiered governments, private sector businesses, and civil society to participate in ecological conservation (LSDRC 2022). For example, if a development project causes a GEP decrease that cannot be offset on-site, the developers would be compelled to pay a “Two-Mountain Company (TMC)”—a new type of firm that has a business model predicated on supplying ecosystem services through GEP—for off-site ecosystem restoration to make up for the original GEP decrement.

#### Key lessons learned

Using GEP as an administrative evaluation indicator can strongly incentivize public agencies to carry out resource conservation, green industrial activities, and sustainable project investments, while also more generally incentivizing ecosystem protection and restoration efforts. Adopting GEP performance evaluation has also promoted improvements in environmental monitoring to inform GEP accounting, so progress and shortcomings are more accurately captured (LSDRC 2022).

### Improving eco-compensation policy

GEP can help address two major difficulties in the implementation of eco-compensation (i.e., public payment for ecosystem services policies), including in China: the determination of compensation standards and benefits accounting and, relatedly, the reasonable distribution of compensation funds (i.e., the amount paid is commensurate with the value of services provisioned).

#### GEP application mechanism

Usually, ecosystem service providers and beneficiaries are involved in eco-compensation policies. Based on ecosystem services flow accounting in GEP, the changes in the value of ecosystem service use resulting from eco-compensation provide important information on policy standards and benefits evaluation (Fig. 6b). For example, the costs and benefits of the "paddy-to-dry" eco-compensation policy in Beijing’s Miyun Reservoir Basin were effectively accounted by considering the flow from the suppliers to the beneficiaries of the water supply and purification services (Zheng et al. 2013). In addition, ecosystem asset accounting in GEP can help optimize the allocation of eco-compensation funds, based on the proportional relationship between GEP values generated by ecosystem assets in different administrative units. This was done in Ezhou City, where GEP adoption helped to more rigorously allocate eco-compensation funds to improve ecosystem services provision (Zhou et al. 2019).

#### Effectiveness

GEP accounting measures both ecosystem assets as stocks and ecosystem services as flows. This approach significantly increases the operability of eco-compensation policies by quantifying the benefits of eco-compensation policies based on the values of ecosystem service use and by scientifically allocating eco-compensation funds based on the proportional relationship of GEP generated by different ecosystem asset units.

#### Key lessons learned

GEP accounting can quantitatively and effectively link ecosystem service providers and beneficiaries, providing a more rigorous basis for determining eco-compensation standards and benefits, and the allocation funds. In addition, clarifying the rights of ownership, use, and operation of ecosystem assets is needed before eco-compensation fund allocation.

### Developing a GEP-based financing mechanism

The construction of financial mechanisms related to GEP can be carried out based on ecosystem assets and GEP, namely ecosystem-asset-based financing mechanism (Fig. 6c) and GEP-based financing mechanism (Fig. 6d).

#### GEP application mechanism

Usually, ecosystem asset owners, investors, governments, and banks are involved in ecosystem-asset-based financing mechanism (Fig. 6c). After the registration and certification of ecosystem assets that clarify the rights of ownership, use, and operation, ecosystem assets can then by transacted through the Operation and Management Platform of Ecosystem Assets built by local commercial banks.^4^ The scattered ecosystem assets from local owners are transformed into contiguous and high-quality ecosystem asset packages, which can attract investors and incentivize greater investment in related green industries (Li et al. 2020; Gao et al. 2022). Using GEP as a benchmark for investments in related industries such as eco-tourism and eco-agriculture is another important policy pathway (Fig. 6d). For example, some regions have established platforms, which are called “Two-Mountains Banks,” for the integration, improvement, management, and market-based trading of ecosystem assets (e.g., property rights for forest stands) and ecological equities (e.g., permits for carbon and pollutant emissions, which correspond to the ecosystem services of carbon sequestration and water purification, respectively) to further the translation of ecological benefits into economic benefits. Two-Mountain Banks use GEP accounting to release “GEP loans” with low interest rates and relatively fast approvals by commercial banks. These GEP loans put future GEP revenues (e.g., from payment for ecosystem services, eco-tourism, eco-agriculture, renewable energy, water conservation, soil conservation, flood control, air purification, water quality purification, and carbon sequestration) as collateral and are used to fast-track green development projects for implementation (Lan and Liu 2022).

#### Effectiveness

Twelve new Two-Mountain Banks have been established in Lishui between 2020 and 2021. They have launched a growing number of GEP loans that exceeded 19 billion RMB (approximately 2.9 billion USD at year-end exchange rates) in 2020 alone (Lan and Liu 2022). Based on the required increment of GEP growth, corresponding GEP loans were used to support ecological industries (e.g., eco-tourism, organic agriculture) and advance green development. To date, as measured in the amount of investment facilitated, this is one of the most effective green financing programs for natural capital in the world.

#### Key lessons learned

The Operation and Management Platform of Ecosystem Assets and Two-Mountain Banks with GEP loans facilitated the transformation of scatted ecosystem assets into contiguous and high-quality ecosystem asset packages, from potential and future ecological benefits to immediate economic benefits. These transformations not only help “green” local economic development, but also strengthen the protection and restoration of local ecosystems.

### Establishing a GEP-based procurement system

GEP-based government procurement refers to the use of various types of funds by different levels of governments and their constituent departments (within a given administrative jurisdiction) to purchase ecosystem services from enterprises, which collect and manage the ecosystem assets from rural collective organizations, other groups or even individuals, and which then share out the economic gains with them (Fig. 6e). This policy is used to leverage social capital to strengthen natural capital and thereby promote green development.

#### GEP application mechanism

Ecosystem service providers, beneficiaries, enterprises, and governments are usually all involved in a GEP-based procurement system (Fig. 6e). General operation of this system is as follows: a "Two-Mountains Company” is established with investment from the ecosystem service providers or ecosystem asset owners. These companies are responsible for the protection and restoration of ecosystem assets, and each owner receives dividends based on the benchmark year’s GEP. Next, the government integrates eco-compensation and ecosystem restoration project funds to establish a “government procurement special fund for ecosystem services.” Based on annual GEP accounting results, the annual increment of GEP is purchased by the government from the Two-Mountains Company. Finally, the Two-Mountains Company invests the procurement funds in ecological conservation and infrastructure projects, with the further aim of enhancing the growth of nature-based industries and green investments (Lan and Liu 2022).

#### Effectiveness

Many regions (e.g., Yunhe County, Jingning She Autonomous County, and Lishui City) have stipulated procurement funds as determined by annual GEP increment (0.1–2%) (Lan and Liu 2022). This public–private partnership approach can boost rural employment and income while improving ecosystem protection and restoration.

#### Key lessons learned

This model can be summarized as "government guides, business engages, land stewards participate, finance is mobilized, and credit is guaranteed." It has demonstrated the ability to incentivize ecosystem protection and restoration and increase income and employment for rural communities.

### Establishing a GEP credit system

GEP credit refers to regulations (and corresponding social obligations) by which individuals and organizations in a certain jurisdiction must abide, so to maintain and enhance GEP. Individuals or organizations can also receive corresponding credit rewards or punishments in terms of credit performance and evaluation.

#### GEP application mechanism

Many stakeholders, from individuals to villages to enterprises, alongside the government, are involved in a GEP credit system (Fig. 6f). First, a GEP credit system incentivizes and punishes the activities of different stakeholders with respect to changes in the local natural capital base. Then, a credit financing model is established by the government in which commercial banks provide collateral-free and low-interest financial support for the rural land stewards who provided public-goods ecosystem services (namely regulating services), with GEP revenues used as collateral (Lan and Liu 2022).

#### Effectiveness

Lishui in Zhejiang Province has established a GEP credit system in which forty-nine positive and negative activities are stipulated, including detailed standards for individuals, enterprises, and villages. Furthermore, 30 rewards (e.g., applying loan with low interest rate) and punishment (e.g., reducing financial subsidies) measures and 35 incentive measures (e.g., increasing credit score through afforestation or green travel) are also stipulated, clarifying which activities are encouraged and which discouraged with respect to GEP (Lan and Liu 2022).

#### Key lessons learned

A GEP credit system incentivizes the participation of local stakeholders in GEP-positive activities, helping to promote society-wide behaviors and norms that are beneficial to nature.

## Future prospects

GEP accounting and its policy applications are at an early stage of development, although pilots and practical explorations have been broadly conducted in China and several other countries. GEP accounting will likely take some years to reach maturity, and even then may not capture the full benefits nature provides to people. The development of GEP has been aided by the extensive work that has already been advanced by SEEA and related efforts. However, many challenges remain in its ongoing development. Going forward, researchers and practitioners should pay closer attention to standardizing methodologies and tools, using more spatially explicit data, investigating ways to incorporate more services [such as the health benefits of nature experience and contact, via many causal pathways (Bratman et al. 2019; Remme et al. 2021)] and deriving insights from more diverse policy applications. In the case of intangible cultural services (including esthetic, spiritual, and other values), appropriate policies can be advanced alongside GEP even in the absence of a meaningful quantification of the services (Satz et al. 2013; IPBES 2022).

### Standardizing methodologies for GEP accounting

Given GEP’s growing appeal and adoption, there is an urgent need to standardize methods to ensure compatibility and comparability (Zhang et al. 2010; Polasky et al. 2015; Jiang et al. 2021a). This could build upon ongoing work by the United Nations in developing international standards for a system of environmental-economic accounts (UN 2021). In addition, compiling the full suite ecosystem services that comprise GEP requires the use of multiple biophysical models. Currently, most existing models do not integrate the location of beneficiaries, which influences the flow of ecosystem services and the quantification of ecosystem service use (Wang et al. 2022). Beneficiaries’ locations and preferences are essential information for applying GEP accounting in a policy context but are often overlooked (Vigl et al. 2017; Dolan et al. 2021). Furthermore, for many ecosystem services, there are large gaps between where ecological modeling stops (e.g., the amount of nutrients in water supply) and where the valuation of ecosystem services begins (e.g., human health impacts). Advances in process-based ecological flow models that incorporate social information (Dolan et al. 2021), integrated ecological-economic modeling focused on tracking the cause and effect of human actions on ecological change and the services thereby provisioned (and ultimately the impacts on human well-being) can help close these gaps (Keeler et al. 2012; Olander et al. 2018).

### Identifying high-quality parameters for GEP accounting

Compiling the full suite of ecosystem services for GEP accounting requires substantial data. The availability of quality data is an important precondition for analysis. High-quality, spatially explicit data not only increase the credibility of GEP accounting, but also can also more detailed, policy-relevant information. However, some technical and institutional challenges remain. For instance, the diversity of ecosystems presents a challenge to the selection of indicators for different ecosystem types and conditions (Hein et al. 2020). Additionally, data from different agencies and organizations may be incompatible, or there may be reluctance to share relevant information among them (Hein et al. 2020). To improve GEP accounting and ensure its reliability for policy, studies should be conducted to address technical issues such as the definition of metrics of ecosystem condition and the capacity of ecosystems to supply services. Scientific efforts relevant to GEP accounting, such as the Earth Observation for Ecosystem Accounting initiative by the Group on Earth Observations, machine-learning techniques, and the utilization of “big data” are currently being developed (Hein et al. 2020). Going forward, identifying the biophysical monitoring necessary to underpin accurate estimates of ecosystem services capacity and the economic methods necessary to inform accurate estimates of prices is crucial. In particular, the availability of relevant data can be continuously improved through the improvement of ecosystem monitoring networks (Hein et al. 2015; Baveye 2017).

### Advancing GEP application in diverse policy contexts

Identifying and institutionalizing nature’s contribution to people can help mainstream ecosystem services into policy and practice (Diaz et al. 2018). Looking ahead, three applications stand out as crucial: (i) continuing to expand how the values of nature are integrated into planning through GEP and its policy applications; (ii) activating more people as catalysts of innovation and social change; and (iii) launching more compelling demonstrations, yielding win–win regeneration of nature through inclusive development pathways (Daily 2021). GEP accounting applications have been deployed in increasingly diverse settings to answer policy-oriented questions. This can help reach the broader community of policy-makers, private sector stakeholders, and civil society actors beyond academia (Comte et al. 2022). Further steps should be taken to strengthen GEP’s transdisciplinary appeal and utility.

In summary, GEP provides a measure of the aggregate monetary value of ecosystem service use in a given area and over a specific accounting period. GEP accounts for ecosystems’ contribution to human well-being and translates those contributions to the economy into monetary terms. Complete GEP accounting, including ecosystem asset stock accounting, ecosystem service supply accounting, ecosystem service use accounting, and the pricing of utilized ecosystem services for GEP aggregation, outlines a supply chain generated by the stewardship of natural capital. Furthermore, GEP accounting provides transparent, trackable, and readily understandable information for decision-makers. Based on these advances, there have been a series of policy innovations across China in recent years, including the development of a GEP-based administrative evaluation system, eco-compensation policies, a GEP-based procurement system, GEP-based financing mechanisms, and a GEP credit system. As a next step, standardizing methodologies and using more spatially explicit data for GEP accounting, as well as advancing GEP application in diverse decision contexts, should be strengthened to better support green and inclusive development across the world.

## Footnotes

1 http://gi.mnr.gov.cn/202004/t20200427_2510189.html.

2 http://www.gov.cn/xinwen/2021-03/24/content_5595373.htm.

3 https://www.sztv.com.cn/ysz/zx/zw/78752890.shtml.

4 http://www.zgnfstcp.com/page/gpxm_type.

## References

[CR1] Arkema KK, Verutes GM, Wood SA, Rosado S, Canto M, Rosenthal A, Ruckelshaus M, Guannel G, Toft J, Faries J, Silver JM, Griffin R, Guerry AD (2015). Embedding ecosystem services in coastal planning leads to better outcomes for people and nature. Proceedings of the National Academy of Sciences of the United States of America.

[CR2] Arnold JG, Moriasi DN, Gassman PW, Abbaspour KC, White MJ, Srinivasan R, Santhi C, Harmel RD, Van Griensven A, Van Liew MW, Kannan N, Jha MK (2012). SWAT: Model use, calibration, and validation. Transactions of the ASABE.

[CR3] Arrow KJ, Dasgupta P, Goulder LH, Mumford KJ, Oleson K (2012). Sustainability and the measurement of wealth. Environment and Development Economics.

[CR4] Banerjee O, Cicowiez M, Horridge M, Vargas R (2019). Evaluating synergies and trade-offs in achieving the SDGs of zero hunger and clean water and sanitation: An application of the IEEM Platform to Guatemala. Ecological Economics.

[CR5] Banerjee O, Cicowiez M, Vargas R, Horridge M (2019). The SEEA-based integrated economic-environmental modelling framework: An illustration with Guatemala’s forest and fuelwood sector. Environmental & Resource Economics.

[CR6] Baveye PC (2017). Quantification of ecosystem services: Beyond all the "guesstimates", how do we get real data?. Ecosystem Services.

[CR7] Bo WJ, Wang LY, Cao JH, Wang XK, Xiao Y, Ouyang ZY (2017). Valuation of China’s ecological assets in forest. Acta Ecologica Sinica.

[CR8] Bratman GN, Anderson CB, Berman MG, Cochran B, de Vries S, Flandersc J, Folke C, Frumkin H, Gross JJ, Hartig T, Kahn PH, Kuo M, Lawler JJ, Levin PS, Lindahl T, Meyer-Lindenberg A, Mitchell R, Ouyang ZY, Roe J, Scarlett L, Smith J, van den Bosch M, Wheeler B, White M, Zheng H, Daily GC (2019). Nature and mental health: An ecosystem service perspective. Science Advances.

[CR9] Breeze TD, Gallai N, Garibaldi LA, Li XS (2016). Economic measures of pollination services: Shortcomings and future directions. Trends in Ecology & Evolution.

[CR10] Chaplin-Kramer R, Sharp RP, Weil C, Bennett EM, Pascual U, Arkema KK, Brauman KA, Bryant BP, Guerry AD, Haddad NM, Hamel P, Hamann M, Johnson JA, Lisa M, Pereira HM, Polasky S, Ruckelshaus M, Shaw MR, Silver JM, Vogl AL, Daily GC (2019). Global modeling of nature’s contributions to people. Science.

[CR11] Cleveland CJ, Betke M, Federico P, Frank JD, Hallam TG, Horn J, López JD, McCracken GF, Medellín RA, Moreno-Valdez A, Sansone CG, Westbrook JK, Kunz TH (2006). Economic value of the pest control service provided by Brazilian free-tailed bats in south-central Texas. Frontiers in Ecology and the Environment.

[CR12] Comte A, Campagne CS, Lange S, Bruzón AG, Hein L, Santos-Martín F, Levrel H (2022). Ecosystem accounting: Past scientific development and future challenges. Ecosystem Services.

[CR13] D’Amato D, Bartkowski B, Droste N (2020). Reviewing the interface of bioeconomy and ecosystem service research. Ambio.

[CR14] Daily GC (2021). The next steps for valuing nature in decision making. Environment: Science and Policy for Sustainable Development.

[CR15] Daily GC, Ruckelshaus M (2022). 25 years of valuing ecosystems in decisions. Nature.

[CR16] Davidson NC, van Dam AA, Finlayson CM, McInnes RJ (2019). Worth of wetlands: Revised global monetary values of coastal and inland wetland ecosystem services. Marine & Freshwater Research.

[CR17] de Groot R, Brander L, van der Ploeg S, Costanza R, Bernard F, Braat L, Christie M, Crossman N, Ghermandi A, Hein L, Hussain S, Kumar P, McVittie A, Portela R, Rodriguez LC, ten Brink P, van Beukering P (2012). Global estimates of the value of ecosystems and their services in monetary units. Ecosystem Services.

[CR18] Diaz S, Pascual U, Stenseke M, Martin-Lopez B, Watson RT, Molnar Z, Hill R, Chan KMA (2018). Assessing nature’s contributions to people. Science.

[CR19] Ding ZW, Zheng H, Wang J, O'Connor P, Li C, Chen XD, Li RD, Ouyang ZY (2022). Integrating top-down and bottom-up approaches improves practicality and efficiency of large-scale ecological restoration planning: Insights from a social-ecological system. Engineering.

[CR20] Dolan R, Bullock JM, Jones JPG, Athanasiadis IN, Martinez-Lopez J, Willcock S (2021). The flows of nature to people, and of people to nature: Applying movement concepts to ecosystem services. Land.

[CR21] Freeman AM, Boucher F, Brockett CD, Cornia GA, Jolly R, Stewart F, Mattuella JL, Lanzer EA (2014). The Measurement of Environmental and Resource Values: Theory and Methods.

[CR22] Gao XL, Yan ZQ, Xin ZR, Kong XS, Xu WH, Zheng H, Ouyang ZY (2022). A study on wetland mitigation banking and solution for localization. Wetland Science and Management.

[CR23] Goulder LH, Kennedy D, Daily GC (1997). Valuing ecosystem services: Philosophical bases and empirical methods. Nature’s Services: Societal Dependence on Natural Ecosystems.

[CR24] Gret-Regamey A, Weibel B (2020). Global assessment of mountain ecosystem services using earth observation data. Ecosystem Services.

[CR25] Hamilton K, Clemens M (1999). Genuine saving rates in developing countries. World Bank Economic Review.

[CR26] Hein L, Obst C, Edens B, Remme RP (2015). Progress and challenges in the development of ecosystem accounting as a tool to analyse ecosystem capital. Current Opinion in Environmental Sustainability.

[CR27] Hein L, Bagstad KJ, Obst C, Edens B, Schenau S, Castillo G, Soulard F, Brown C (2020). Progress in natural capital accounting for ecosystems. Science.

[CR28] Huang BB, Li RN, Ding ZW, O’Connor P, Kong LQ, Xiao Y, Xu WH, Guo YN (2020). A new remote-sensing-based indicator for integrating quantity and quality attributes to assess the dynamics of ecosystem assets. Global Ecology and Conservation.

[CR29] IPCC (Intergovernmental Panel on Climate Change), Geneva. 2018. *Global Warming of 1.5°C: An IPCC Special Report on the Impacts of Global Warming of 1.5°C Above Pre-Industrial Levels and Related Global Greenhouse Gas Emission Pathways, in the Context of Strengthening the Global Response to the Threat of Climate Change, Sustainable Development, and Efforts to Eradicate Poverty.*

[CR30] IPBES (Intergovernmental Science-Policy Platform on Biodiversity and Ecosystem Services). 2019. *The Global Assessment Report on Biodiversity and Ecosystem Services: Summary for Policymakers.*

[CR31] IPBES. 2022. *Methodological Assessment Report on the Diverse Values and Valuation of Nature of the Intergovernmental Science-Policy Platform on Biodiversity and Ecosystem Services.* Balvanera, P., U. Pascual, M. Christie, B. Baptiste, and D. González-Jiménez. IPBES secretariat, Bonn, Germany. 10.5281/zenodo.6522522.

[CR32] IUCN. 2019. *The IUCN Red List of Threatened Species.* https://www.iucnredlist.org. Accessed 10 Feb 2023.

[CR33] Jiang HQ, Wu WJ, Wang JN, Yang WS, Gao YM, Duan Y, Ma GX, Wu CS, Shao JC (2021). Mapping global value of terrestrial ecosystem services by countries. Ecosystem Services.

[CR34] Jiang W, Wu T, Fu BJ (2021). The value of ecosystem services in China: A systematic review for twenty years. Ecosystem Services.

[CR35] Karp DS, Mendenhall CD, Sandí RF, Ehrlich PR, Hadly EA, Daily GC (2013). Forest bolsters birds, pest control, and coffee yield. Ecology Letters.

[CR36] Keeler BL, Polasky S, Brauman KA, Johnson KA, Finlay JC, O'Neill A, Kovacs K, Dalzell B (2012). Linking water quality and well-being for improved assessment and valuation of ecosystem services. Proceedings of the National Academy of Sciences of the United States of America.

[CR37] Kimmerer RW (2013). Braiding Sweetgrass: Indigenous Wisdom, Scientific Knowledge, and the Teachings of Plants.

[CR38] Lan, J.P., and K.Q. Liu. 2022. Innovation and practice of GEP application in Lishui. https://www.zjskw.gov.cn/art/2022/1/25/art_1229556991_44789.html. Accessed 10 April 2022

[CR39] Li HW, Bo F, Cui L (2020). Theoretical innovation and practical exploration for value realization of ecosystem services. Governance Studies.

[CR40] LSDRC (Lishui Development and Reform Committee). 2022. *Establishing “six entrance” system and pushing policy application of GEP accounting in Lishui.* http://fgw.lishui.gov.cn/art/2021/1/21/art_1229278588_58720103.html. Accessed 10 Feb 2023.

[CR41] MA (Millennium Ecosystem Assessment) (2005). Ecosystems and Human Well-Being: Synthesis.

[CR42] Ma GX, Zhao XT, Wu Q, Pan T (2015). Concept definition and system construction of gross ecosystem product. Resources Science.

[CR43] Maes J, Teller A, Erhard M, Grizzetti B, Barredo JI, Paracchini ML, Condé S, Somma F (2018). Mapping and Assessment of Ecosystems and Their Services: An Analytical Framework for Ecosystem Condition.

[CR44] Managi S, Kumar P (2018). Inclusive Wealth Report 2018: Measuring Progress Toward Sustainability.

[CR45] Mandle L, Ouyang ZY, Salzman J, Daily GC (2019). Green Growth That Works Natural Capital Policy and Finance Mechanisms from Around the World: Natural Capital Policy and Finance Mechanisms from Around the World.

[CR46] Mandle L, Mitchell MG, Bremer LL, Gourevitch JD, Hawthorne P, Johnson JA, Robinson BE, Smith JR, Sonter LJ, Verutes GM, Vogl AL, Daily GC, Ricketts TH (2021). Increasing decision relevance of ecosystem service science. Nature Sustainability.

[CR47] Mastrángelo ME, Pérez-Harguindeguy N, Enrico L, Bennett E, Lavorel S, Cumming GS, Abeygunawardane D, Amarilla LD, Burkhard B, Egoh BN, Frishkoff L, Galetto L, Huber S, Karp DS, Ke A, Kowaljow E, Locatelli B, Meyfroidt P, Mwampamba TH, Nel J, Nicholas KA, Nicholson C, Oteros-Rozas E, Rahlao SJ, Raudsepp-Hearne C, Ricketts T, Shrestha UB, Torres C, Winkler KJ, Zoeller K (2019). Key knowledge gaps to achieve global sustainability goals. Nature Sustainability.

[CR48] Matias DMS, Leventon J, Rau AL, Borgemeister C, von Wehrden H (2017). A review of ecosystem service benefits from wild bees across social contexts. Ambio.

[CR49] NDRC (National Development and Reform Committee of China), and NSB (National Statistics Bureau of China) (2022). Accounting Specification of Gross Ecosystem Product.

[CR50] Olander L, Polasky S, Kagan JS, Johnston RJ, Wainger L, Saah D, Maguire L, Boyd J (2017). So you want your research to be relevant? Building the bridge between ecosystem services research and practice. Ecosystem Services.

[CR51] Olander L, Johnston RJ, Tallis H, Kagan J, Maguire LA, Polasky S, Urban D, Boyd J (2018). Benefit relevant indicators: Ecosystem services measures that link ecological and social outcomes. Ecological Indicators.

[CR52] Ouyang ZY, Jin LS (2017). Developing Gross Ecosystem Product and Ecological Asset Ac-counting for Eco-compensation.

[CR53] Ouyang ZY, Zhu C, Yang G, Xu W, Zheng H, Zhang Y, Xiao Y (2013). Gross ecosystem product: Concept, accounting framework and case study. Acta Ecologica Sinica.

[CR54] Ouyang ZY, Zheng H, Xiao Y, Polasky S, Liu J, Xu W, Wang Q, Zhang L, Xiao Y, Rao E, Jiang L, Lu F, Wang X, Yang G, Gong S, Wu B, Zeng Y, Yang W, Daily GC (2016). Improvements in ecosystem services from investments in natural capital. Science.

[CR55] Ouyang ZY, Zheng H, Xie G, Yang W, Liu JG, Shi Y, Yang D (2016). Accounting theories and technologies for ecological assets, ecological compensation and scientific and technological contribution to ecological civilization. Acta Ecologica Sinica.

[CR56] Ouyang ZY, Song CS, Zheng H, Polasky S, Xiao Y, Bateman IJ, Liu JG, Ruckelshaus M, Shi FQ, Xiao Y, Xu WH, Zou ZY, Daily GC (2020). Using gross ecosystem product (GEP) to value nature in decision making. Proceedings of the National Academy of Sciences of the United States of America.

[CR57] Polasky S, Bryant B, Hawthorne P, Johnson J, Keeler B, Pennington D (2015). Inclusive wealth as a metric of sustainable development. Annual Review of Environment and Resources.

[CR58] Polasky S, Kling CL, Levin SA, Carpenter SR, Daily GC, Ehrlich PR, Heal GM, Lubchenco J (2019). Role of economics in analyzing the environment and sustainable development. Proceedings of the National Academy of Sciences of the United States of America.

[CR59] Rao NS, Ghermandi A, Portela R, Wang XW (2015). Global values of coastal ecosystem services: A spatial economic analysis of shoreline protection values. Ecosystem Services.

[CR60] Remme RP, Frumkin H, Guerry AD, King AC, Mandle L, Sarabu C, Bratman GN, Giles-Corti B, Hamel P, Han B, Hicks JL, James P, Lawler JJ, Lindahl T, Liu H, Lu Y, Oosterbroek B, Paudel B, Sallis JF, Schipperijn J, Sosič R, de Vries S, Wheeler BW, Wood SA, Wu T, Daily GC (2021). An ecosystem service perspective on urban nature, physical activity, and health. Proceedings of the National Academy of Sciences of the United States of America.

[CR61] Reynaud A, Lanzanova D (2017). A global meta-analysis of the value of ecosystem services provided by lakes. Ecological Economics.

[CR62] Ricketts TH, Daily GC, Ehrlich PR, Michener C (2004). Economic value of tropical forest to coffee production. Proceedings of the National Academy of Sciences of the United States of America.

[CR63] Rieb JT, Daily GC, Armsworth PR, Bonn A, Cumming GS, Eigenbrod F, Grimm V, Jackson BM, Marques A, Pattanayak SK, Pereira HM, Peterson GD, Ricketts TH, Robinson BE, Schröter M, Schulte LA, Seppelt R, Turner MG, Bennett EM (2017). When, where and how nature matters for ecosystem services: Challenges for the next generation of ecosystem service models. BioScience.

[CR64] Rockström J, Steffen W, Noone K, Persson Å, Chapin FS, Lambin EF, Lenton TM, Scheffer M, Folke C, Schellnhuber HJ, Nykvist B, Wit CA, Hughes T, Leeuw S, Rodhe H, Sörlin S, Snyder PK, Costanza R, Svedin U, Falkenmark M, Karlberg L, Corell RW, Fabry VJ, Hansen J, Walker B, Liverman D, Richardson K, Crutzen P, Foley JA (2009). A safe operating space for humanity. Nature.

[CR65] Satz D, Gould RK, Chan KMA, Guerry A, Norton B, Satterfield T, Halpern BS, Levine J, Woodside U, Hannahs N, Basurto X, Klain S (2013). The challenges of incorporating cultural ecosystem services into environmental assessment. Ambio.

[CR66] Sharp R, Chaplin-Kramer R, Wood S, Guerry A, Douglass J (2018). InVEST User’s Guide.

[CR67] Song C, Xiao Y, Bo W, Xiao Y, Zou Z, Ouyang ZY (2019). The ecological asset accounting method study: A case study of Qinghai Province. Acta Ecologica Sinica.

[CR68] Stiglitz J, Sen A, Fitoussi JP (2010). Mis-Measuring Our Lives: Why GDP Doesn’t Add Up.

[CR69] Taye FA, Folkersen MV, Fleming CM, Buckwell A, Mackey B, Diwakar KC, Le D, Hasan S, Ange CS (2021). The economic values of global forest ecosystem services: A meta-analysis. Ecological Economics.

[CR70] The Chinese Government. 2015. *Central Committee of the CPC and the State Council of China, Comprehensive Pro-gram for Reform of the Ecological Progress System.* http://english.www.gov.cn/archive/state_council_gazette/2015/10/10/content_281475208414884.htm. Accessed 7 Dec 2017.

[CR71] Turner RK, Daily GC (2008). The ecosystem services framework and natural capital conservation. Environmental and Resource Economics.

[CR74] United Nations, European Commission, Food and Agriculture Organization, International Monetary Fund, Organization for Economic Cooperation and Development, and World Bank (2012). System of Environmental-Economic Accounting 2012: Central Framework.

[CR75] United Nations, European Commission, Food and Agriculture Organization, In-ternational Monetary Fund, Organization for Economic Cooperation and Develop-ment, and World Bank (2013). System of Environmental-Economic Accounting 2012: Experimental Ecosystem Accounting.

[CR72] United Nations University, The International Human Dimensions Programme, and United Nations Environment Programme (2012). Inclusive Wealth Report 2014: Measuring Progress Toward Sustainability.

[CR73] United Nations University, The International Human Dimensions Programme on Global Environmental Change, and United Nations Environment Programme (2014). Inclusive Wealth Report 2014: Measuring Progress Toward Sustainability.

[CR76] UN (United Nations). 2021. *System of Environmental-Economic Accounting- Ecosystem Accounting (SEEA- EA). White cover publication, pre-edited text subject to official editing.* https://seea.un.org/ecosystem-accounting. Accessed 10 Feb 2023.

[CR77] Vackaru D, Grammatikopoulou I (2019). Toward development of ecosystem asset accounts at the national level. Ecosystem Health and Sustainability.

[CR78] Vallecillo S, Kakoulaki G, Notte AL, Feyen L, Dottori F, Maes J (2020). Accounting for changes in flood control delivered by ecosystems at the EU level. Ecosystem Services.

[CR79] Van Wensem J, Calow P, Dollacker A, Maltby L, Van Houtven G (2017). Identifying and assessing the application of ecosystem services approaches in environmental policies and decision making. Integrated Environmental Assessment and Management.

[CR80] Vári Á, Kozma Z, Pataki B, Jolánkai Z, Kardos M, Decsi B, Pinke Z, Jolánkai G, Pásztor L, Condé S, Sonderegger G, Czúcz B (2022). Disentangling the ecosystem service ‘flood regulation’: Mechanisms and relevant ecosystem condition characteristics. Ambio.

[CR81] Vigl LE, Depellegrin D, Pereira P, de Groot R, Tappeiner U (2017). Mapping the ecosystem service delivery chain: Capacity, flow, and demand pertaining to aesthetic experiences in mountain landscapes. Science of the Total Environment.

[CR82] Wang LJ, Zheng H, Chen YZ, Ouyang ZY, Hu XF (2022). Systematic review of ecosystem services flow measurement: Main concepts, methods, applications and future directions. Ecosystem Services.

[CR83] World Bank (2006). Where is the Wealth of Nations?.

[CR84] World Bank (2011). The Changing Wealth of Nations: Measuring Sustainable Development in the New Millennium.

[CR85] World Bank (2021). The Changing Wealth of Nations 2021: Managing Assets for the Future.

[CR86] World Bank. 2023. https://data.worldbank.org/indicator/NY.GDP.MKTP.KD. Accessed 9 Sept 2023.

[CR87] Zhang BA, Li WH, Xie GD (2010). Ecosystem services research in China: Progress and perspective. Ecological Economics.

[CR88] Zhang H, Garratt MPD, Bailey A, Potts SG, Breeze T (2018). Economic valuation of natural pest control of the summer grain aphid in wheat in South East England. Ecosystem Services.

[CR89] Zheng H, Robinson BE, Liang YC, Polasky S, Ma DC, Wang FC, Ruckelshaus M, Ouyang ZY, Daily GC (2013). The benefits, costs, and livelihood implications of a regional payment for ecosystem service program. Proceedings of the National Academy of Sciences of the United States of America.

[CR90] Zhou YJ, Zhou JX, Liu HL, Xia M (2019). Study on eco-compensation standard for adjacent administrative districts based on the maximum entropy production. Journal of Cleaner Production.

